# Underexpression of peroxisome proliferator-activated receptor (PPAR)*γ* in PAX8/PPAR*γ*-negative thyroid tumours

**DOI:** 10.1038/sj.bjc.6601989

**Published:** 2004-07-06

**Authors:** A R Marques, C Espadinha, M J Frias, L Roque, A L Catarino, L G Sobrinho, V Leite

**Affiliations:** 1 Centro de Investigação de Patobiologia Molecular, Instituto Português de Oncologia Francisco Gentil, Rua Professor Lima Basto, 1099-023 Lisboa Codex, Portugal; 2 Departamento de Patologia Morfológica; Instituto Português de Oncologia Francisco Gentil, Rua Professor Lima Basto, 1099-023 Lisboa Codex, Portugal

**Keywords:** PPAR*γ*, PAX8, underexpression, follicular thyroid tumours

## Abstract

The expression of peroxisome proliferator-activated receptor (PPAR)*γ* in thyroid neoplasias and in normal thyroid (NT) tissues has not been fully investigated. The objectives of the present work were: to study and compare the relative expression of PPAR*γ* in normal, benign and malignant thyroid tissues and to correlate PPAR*γ* immunostaining with clinical/pathological features of patients with thyroid cancer. We analysed the expression of PPAR*γ* in several types of thyroid tissues by reverse transcription–polymerase chain reaction (RT–PCR), interphase fluorescent *in situ* hybridisation, real-time RT–PCR and immunohistochemistry. We have demonstrated that NT tissues express PPAR*γ* both at mRNA and at protein level. PAX8-PPAR*γ* fusion gene expression was found in 25% (six of 24) of follicular thyroid carcinomas (FTCs) and in 17% (six of 36) of follicular thyroid adenomas, but in none of the 10 normal tissues, 28 nodular hyperplasias, 38 papillary thyroid carcinomas (PTCs) and 11 poorly differentiated thyroid carcinomas (PDTCs). By real-time RT–PCR, we observed that tumours negative for the PAX8-PPAR*γ* rearrangement expressed lower levels of PPAR*γ* mRNA than the NT. Overexpression of PPAR*γ* transcripts was detected in 80% (four of five) of translocation-positive tumours. Diffuse nuclear staining was significantly (*P*<0.05) less prevalent in FTCs (53%; 18 of 34), PTCs (49%; 19 of 39) and PDTCs (0%; zero of 13) than in normal tissue (77%; 36 of 47). Peroxisome proliferator-activated receptor*γ*-negative FTCs were more likely to be locally invasive, to persist after surgery, to metastasise and to have poorly differentiated areas. Papillary thyroid carcinomas with a predominantly follicular pattern were more often PPAR*γ* negative than classic PTCs (80% *vs* 28%; *P*=0.01). Our results demonstrated that PPAR*γ* is underexpressed in translocation-negative thyroid tumours of follicular origin and that a further reduction of PPAR*γ* expression is associated with dedifferentiation at later stages of tumour development and progression.

Peroxisome proliferator-activated receptors (PPARs) are members of the nuclear receptor superfamily, which form heterodimers with retinoid X receptor. The heterodimers activate the transcription of specific genes in response to binding of a ligand. Three PPAR isoforms have been described: *α*, *β* (also called *δ*, NUC-1 or FAAR) and *γ* (Desvergne and Wahli, 1999). PPAR*γ* is the most intensively studied isoform. It has been shown that this nuclear receptor is important in several biological pathways involving cell differentiation, insulin sensitivity, atherosclerosis and cancer (Rosen and Spiegelman, 2001). There are two protein isoforms (PPAR*γ*_1_ and PPAR*γ*_2_) generated by alternative splicing and alternative promoter usage. Peroxisome proliferator-activated receptor*γ*_1_ isoform is encoded by three transcripts, which differ in 5′-untranslated region (variants *γ*_1_, *γ*_3_ and *γ*_4_). Variant *γ*_2_ encodes isoform PPAR*γ*_2_ (Martin *et al*, 1998; Sundvold and Lien, 2001). Peroxisome proliferator-activated receptor*γ*_2_ contains 30 additional amino acids in the N-terminus (Tontonoz *et al*, 1994). Peroxisome proliferator-activated receptor*γ* is activated by natural ligands (fatty acids and eicosanoids) (Chawla and Lazar, 1994; Tontonoz *et al*, 1994; Forman *et al*, 1995; Kliewer *et al*, 1995) and by synthetic ligands (thiazolidinediones) (Lehmann *et al*, 1995). Peroxisome proliferator-activated receptor*γ* activation was reported to inhibit the growth and, in some cases, to induce apoptosis or differentiation of tumour cells from different lineages: liposarcoma (Tontonoz *et al*, 1997; Demetri *et al*, 1999), breast cancer (Elstner *et al*, 1998; Mueller *et al*, 1998), prostate cancer (Kubota *et al*, 1998), colorectal cancer (Brockman *et al*, 1998; Sarraf *et al*, 1998; Kitamura *et al*, 1999), bladder cancer (Guan *et al*, 1999), non-small-cell lung carcinoma (Chang and Szabo, 2000), pancreatic cancer (Motomura *et al*, 2000), gastric cancer (Sato *et al*, 2000), renal carcinoma (Inoue *et al*, 2001), testicular cancer (Hase *et al*, 2002) and liver cancer (Toyoda *et al*, 2002).

Kroll *et al* (2000) reported that t(2;3)(q13;p25), a chromosomal translocation detected in a subset of follicular thyroid carcinomas (FTCs), originates a fusion gene composed by DNA-binding domain of the thyroid transcription factor PAX8 and domains A to F of PPAR*γ*. Recently, our group and others (Marques *et al*, 2002; Nikiforova *et al*, 2002; Cheung *et al*, 2003) have detected the expression of PAX8-PPAR*γ* gene not only in FTCs but also in follicular thyroid adenomas (FTAs). Ohta *et al* (2001) studied the expression of PPAR*γ* in papillary thyroid carcinoma (PTC) cell lines and in thyroid tumours. They showed that most cell lines and half of PTCs expressed PPAR*γ*, while normal adjacent tissue and two FTAs were negative. This group as well as Martelli *et al* (2002) also demonstrated that PPAR*γ* agonists induce apoptosis and inhibit the growth of thyroid tumour cells.

Several studies have demonstrated that, compared to their normal counterparts, the expression of PPAR*γ* in tumour cells is either overexpressed, such as in renal cell carcinoma (Inoue *et al*, 2001) and testicular cancer (Hase *et al*, 2002), underexpressed, such as in oesophageal carcinomas (Terashita *et al*, 2002) or is equal to the normal tissue, such as in colonic adenocarcinomas (Sarraf *et al*, 1998). This last group has also identified somatic mutations of PPAR*γ* in four of 55 sporadic primary colorectal carcinomas (Sarraf *et al*, 1999). The expression of PPAR*γ* in thyroid neoplasias and in the normal thyroid (NT) tissue has not been fully investigated. We have expanded our previous study (Marques *et al*, 2002) and analysed the expression of PPAR*γ* in a series of thyroid tumours and correspondent normal tissue by reverse transcription–polymerase chain reaction (RT–PCR), interphase fluorescent *in situ* hybridisation (FISH), real-time RT–PCR and immunohistochemistry. We observed that PPAR*γ* expression is usually underexpressed in multiple types of thyroid tumours, and that this may be an important event in the development of thyroid neoplasias.

## MATERIALS AND METHODS

### Materials

The number of cases analysed by each technique for the different histological groups is represented in Table 1

**Table 1:** Thyroid tissues analysed for PPAR*γ* expression

	**Techniques**		
**Histology**	**RT–PCR/FISH**	**Real-time RT–PCR**	**PPAR*γ* staining**
Normal thyroid	10	10	47
Nodular hyperplasia	28	0	28
Follicular adenomas	36	13	86
Follicular carcinomas	24	7	34
Papillary carcinomas	38	9	39
Poorly differentiated carcinomas	11	0	13

RT–PCR=reverse transcription–polymerase chain reaction; FISH=fluorescent *in situ* hybridisation; PPAR=peroxisome proliferator-activated receptor.

. Paraffin-embedded tissues and frozen tissues were available in 247 samples and in 131 samples, respectively. Haematoxylin- and eosin-stained sections from each sample were evaluated histologically by two pathologists to classify tumours according to the 1988 World Health Organisation histological classification of thyroid tumours. The extent of papillary carcinomas was classified according to the system of DeGroot *et al* (1990) and the metastasis-age-completeness-of-resection-invasion-size-score (MACIS) (Hay *et al*, 1993). The system of DeGroot *et al* (1990) categorises the patients with PTC by clinical class: I, with intrathyroidal disease; II, with cervical adenopathies; III, with extrathyroidal invasion and IV, with distant metastasis. The prognostic score defined as MACIS was calculated according to Hay *et al* (1993): MACIS=3.1 (if aged ⩽39 years) or 0.08 × age (if aged ⩾40 years), +0.3 × tumour size (in centimetres), +1 (if incompletely resected), +1 (if locally invasive) and +3 (if distant metastasis present).

### RNA extraction, RT–PCR and sequencing

Total RNA was extracted from frozen tumours using TRIzol reagent (Life Technologies, Inc., Gaithersburg, MD, USA), according to the manufacturer's protocol. RNA was quantified by UV spectrophotometry (optical density measured at 260 nm). Complementary DNA (cDNA) was synthesised from 1 *μ*g of RNA at 37°C for 90 min, using oligo-(dT) primers (Life Technologies, Inc.) and reverse transcriptase (Life Technologies, Inc.). PAX8-PPAR*γ* fusion gene expression was analysed by RT–PCR as described previously (Marques *et al*, 2002).

To analyse the expression of the various PPAR*γ* mRNA isoforms, segments from the 5′-terminal region of the PPAR*γ* gene were amplified by PCR using forward primers, located in exons A_1_, A_2_ and B and reverse primers located in exon 1. Primer sequences are presented in Table 2

**Table 2:** RT–PCR oligonucleotide primer sequences

**Primer**	**Exon (PPAR*γ*)**	**Sequence (5′–3′)**
P_1_ (forward)	A_1_	CGGAGCCCGAGCCCGAG
P_2_ (forward)	A_1_	CAGCCGCCGCCTGGGGC
P_3_ (forward)	A_2_	ACACTAAACCACCAATATACAA
P_4_ (forward)	A_2_	CAAGGCCATTTTGTCAAACG
P_5_ (forward)	B	CGGATTGATCTTTTGCTAGAT
P_6_ (forward)	B	GTTATGGGTGAAACTCTGGG
P_7_ (reverse)	1	CAAAGGAGTGGGAGTGGTCT^a^
P_8_ (reverse)	1	CATTACGGAGAGATCCACGGT^a^

RT–PCR=reverse transcription–polymerase chain reaction; PPAR=peroxisome proliferator-activated receptor.

a Kroll *et al* (2000).

. First round amplifications were performed using 1 *μ*l of cDNA, forward primers P_1_, P_3_ and P_5_ and reverse primer P_7_. A measure of 1 *μ*l of each amplification reaction was then used as template for second amplification reactions with nested primers P_2_, P_4_, P_6_ (forward) and P_8_ (reverse). A total of 25 *μ*l reactions were carried out on over 35 cycles using the following conditions: 95°C for 1 min, 55–57°C for 1 min and 72°C for 1 min. Amplification reactions contained final concentrations of 20 mM Tris-HCl (pH 8.4), 50 mM KCl, 200 *μ*M dNTPs (Amersham Pharmacia Biotech, Uppsala, Sweden), 1.0–2.5 mM MgCl_2_, 10 pmol of each primer (forward and reverse) and 1.5 U of *Taq* DNA Polymerase (Life Technologies, Inc.). Negative controls for cDNA synthesis and PCRs, in which the template was replaced by sterile water, were included in each experiment. RNA integrity and efficiency of cDNA synthesis were tested in each sample by performing RT–PCR amplification for the housekeeping gene phosphoglycerate kinase-1 (Sugg *et al*, 1998). Normal colon tissue was used as positive control for the analysis of PPAR*γ* expression (Sarraf *et al*, 1998).

PCR products were analysed and purified by electrophoresis in a 2% agarose gel stained with ethidium bromide. Polymerase chain reaction products were also subjected to automatic sequencing (ABI Prism 310 Genetic Analyser using the ABI Prism Big Dye Terminator Cycle Sequencing Ready Reaction Kit Version 2; Applied Biosystems, PE Corporation, Foster City, CA, USA).

### Interphase FISH analysis

Fluorescent *in situ* hybridisation was performed on isolated nuclei extracted from 50 *μ*m paraffin-embedded sections of thyroid tumours with BAC probes for PPAR*γ* (RPCI 1130 G23, BAC PAC Resources) and PAX8 (RPCI 1165 I12, BAC PAC Resources). Briefly, PPAR*γ* clone DNA was labelled with digoxigenin and PAX8 DNA with biotin by random priming, using the Bioprime DNA labelling system (Invitrogen S.A., Barcelona, Spain). Nuclear suspensions were spotted on SuperFrost slides (Menzel-Glaser, GMbH, Memmert, Germany) and pretreated with 0.1% pepsin (Sigma-Aldrich, St Louis, MO, USA) in 0.2% HCl at 37°C. Probe mixture in 50% formamide in 2 × SSC was codenatured with nuclear DNA at 80°C for 2 min. Detection of the digoxigenin-labelled PPAR*γ* probe was accomplished using an anti-digoxigenin fluorescein antibody (Roche Diagnostics GMbH, Manheim, Germany) and the biotinylated-labelled PAX8 probe with CY_3_–avidin (Jackson Immunoresearch Lab, West Grove, USA). Nuclei were counterstained with DAPI-Vectashield mounting solution (Vector, Burlingame, USA). Fluorescence hybridisation signals were analysed and recorded with a Cytovision System (Applied Imaging, New Castle, UK). For each case 200 intact nonoverlapping nuclei were counted. Nuclei in which the two probes were fused, touched or were close to each other (distance ⩽1 probe signal) were scored as positive for the fusion gene.

### Real-time RT–PCR

The real-time quantitative PCR was performed in a 96-well reaction plate (MicroAmp®Optical 96-Well Reaction Plate, Applied Biosystems, PE Corp.) on an ABI PRISM® 7000 Sequence Detector System (Applied Biosystems, PE Corp.), according to the manufacturer's instructions. TaqMan® One Step PCR Master Mix Reagents Kit (P/N 4309169; Applied Biosystems, PE Corp.) was used to generate fluorescence signals during each PCR cycle. Specific primers and the probe were designed by Pre-Developed Taqman®Assay Reagents (Assays-on-demand, P/N 4331182, Applied Biosystems, PE Corp.). The amplified region contained exons 5 and 6 from PPAR*γ* gene. In order to normalise the differences in the amount of total RNA used in each reaction, we performed the amplification of glyceraldehyde-3-phosphate dehydrogenase (GAPDH) RNA as endogenous control (FG HUMAN GAPDH 0211014, P/N 4333764F, Applied Biosystems, PE Corp.). A pool of five NT tissues was used as calibrator for determining the relative expression of PPAR*γ* gene in the tumour samples as described previously (Lazar *et al*, 1999).

### Immunohistochemistry

Formalin-fixed paraffin-embedded sections (3 *μ*m) were attached to glass slides pretreated with gelatin. The sections were then dried at 37°C overnight and dewaxed with xylol. Endogenous peroxidase was inhibited with 0.6% H_2_O_2_ in methanol for 10 min. Antigen retrieval was performed using a stainless-steel 6-l capacity pressure cooker, with 0.01 M sodium citrate buffer (pH 6.0), for 6 min at full pressure. Slides were incubated with normal goat serum 1 : 10 (DAKO X907, DAKO Corp., Golstrup, Denmark) for 10 min before blocking the endogenous avidin and biotin (Vector SP-2001, Vector Laboratories, Inc., Burlingame, CA, USA). Peroxisome proliferator-activated receptor*γ* primary antibody 1 : 30 (Santa Cruz Biotechnology, Inc., Santa Cruz, CA, USA) was incubated for 30 min. Specificity of PPAR*γ* immunostaining was demonstrated by preincubating the samples with PPAR*γ* blocking peptide 1 : 10 (Santa Cruz Biotechnology, Inc.). Bound primary antibody was detected using biotinylated goat anti-mouse and anti-rabbit immunoglobulin G, being subsequently amplified with streptavidin conjugated to horseradish peroxidase (DAKO K5001; DAKO Corp.). All incubations were performed at room temperature. The peroxidase staining reaction was revealed with a solution containing 3,3′-diaminobenzidine tetrachloride. Sections were counterstained with Mayer's haematoxylin, dehydrated and mounted.

### Statistical analysis

The frequencies of PPAR*γ* transcript variants and the level of PPAR*γ* mRNA in each tumour histotype were analysed by *χ*^2^ test and unpaired *t*-test, respectively. Peroxisome proliferator-activated receptor*γ* mRNA levels in PTCs and in corresponding NT tissues were compared using a paired *t*-test. Peroxisome proliferator-activated receptor*γ* immunostaining for nodular hyperplasias (NH) and thyroid tumours was compared with the staining in NT tissues by two-tailed Fisher's exact test. We also correlated the PPAR*γ* immunostaining in FTCs and PTCs with clinical/pathological features of the patients by unpaired *t*-test, two-tailed Fisher's exact test or *χ*^2^ test as appropriate. *P*-values less than 0.05 were considered significant. Statistical analysis was performed using Graph Pad Prism version 2.0 (San Diego, CA, USA).

## RESULTS

### Analysis of PPAR*γ* transcript variants

RNA from 72 frozen samples (6 normal tissues, 29 FTAs, 9 FTCs, 24 PTCs and four poorly differentiated thyroid carcinomas (PDTCs)) was analysed by RT–PCR. Peroxisome proliferator-activated receptor*γ* transcript variants were determined by combining the RT–PCR results obtained for each primer pair. Peroxisome proliferator-activated receptor*γ*_3_ could be detected only in the five cases that did not present PPAR*γ*_1_, because RT–PCR with primer pairs P_3_P_7_ or P_4_P_8_ originated products with exactly the same size in both variants. Most thyroid tissues expressed PPAR*γ*_1_, PPAR*γ*_2_ and PPAR*γ*_4_, and the proportion of specific variants expressed was similar in NT tissues and in the various types of thyroid tumours (data not shown).

### PAX8-PPAR*γ* fusion gene expression

The fusion gene was detected by RT–PCR and/or interphase FISH analysis. Six out of 24 (25%) FTCs and six out of 36 (17%) FTAs were positive for PAX8-PPAR*γ* fusion gene expression. The rearrangement was not detected in 10 NT tissues, 28 NHs, 38 PTCs and 11 PDTCs.

### Quantitative analysis of PPAR*γ* gene expression

**Figure 1:**
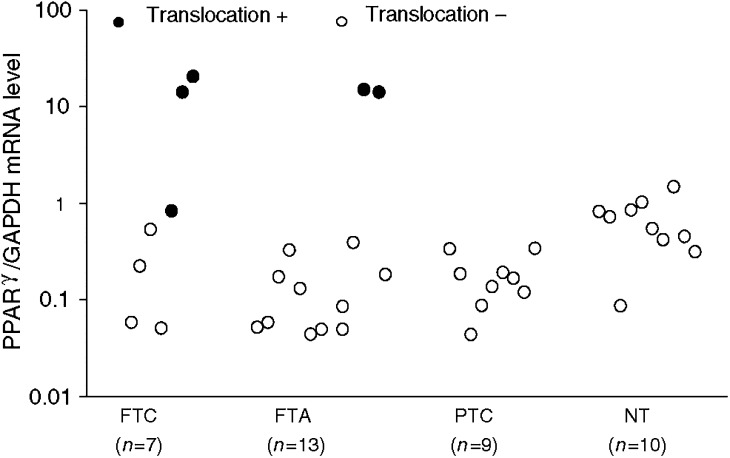
Quantitative analysis of PPAR*γ* expression by real-time RT–PCR in several thyroid samples. The mean expression in negative PAX8-PPAR*γ* follicular tumours (FTA: 0.14±0.22; *P*=0.001 and FTC: 0.22±0.23; *P*=0.05) and in papillary carcinomas (0.18±0.10; *P*=0.002) was lower than in NT tissue (0.68±0.40). PAX8-PPAR*γ*-positive follicular adenomas and carcinomas presented PPAR*γ* mRNA levels that were increased by 22- (14.68±0.67; *P*<0.0001) and 17-fold (11.88±10.09; *P*=0.002) compared to normal tissue (0.68±0.40). One FTC case positive for the fusion gene exhibited a PPAR*γ* mRNA level (0.84) within the normal mean. FTC – follicular thyroid carcinomas; FTA – follicular thyroid adenomas; PTC – papillary thyroid carcinomas, NT – normal tissue.

The mRNA level of PPAR*γ* in thyroid tissues is represented in Figure 1

**Figure 2:**
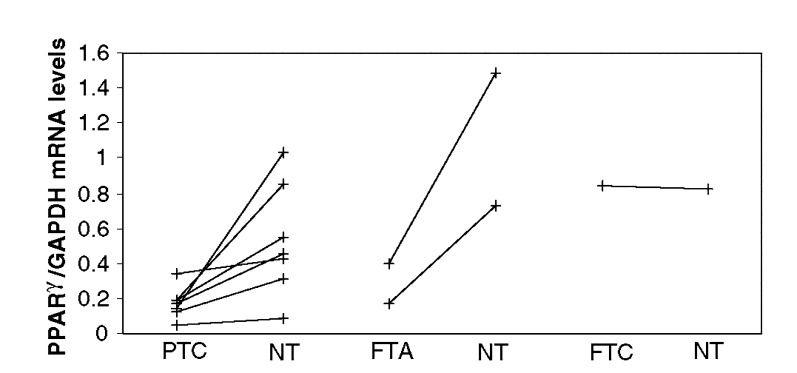
Quantitative analysis of PPAR*γ* mRNA by real-time RT–PCR in thyroid tumours and in the corresponding normal adjacent tissue. The expression level in each PTC was lower than in normal adjacent tissue. The PPAR*γ* mRNA level in two FTAs was also lower than in corresponding normal tissues. One FTC translocation-positive exhibited a ratio similar to its surrounding normal tissue. PTC – papillary thyroid carcinoma; FTA – follicular thyroid adenoma; FTC – follicular thyroid carcinoma; NT – normal thyroid.

. The mean expression level in PAX8-PPAR*γ*-negative FTAs (0.14±0.22; *P*=0.001) and FTCs (0.22±0.23; *P*=0.05) was significantly lower than in normal tissue (0.68±0.40). In contrast, PAX8-PPAR*γ*-positive FTAs and FTCs presented mean PPAR*γ* mRNA levels, which were, respectively, 22- (14.68±0.67; *P*<0.0001) and 17-fold (11.88±10.09; *P*=0.002), higher than the normal mean. However, one FTC case positive for the fusion gene showed a ratio (0.84) within the normal mean (0.68±0.40). We also observed that PTCs showed a ratio (0.18±0.10; *P*=0.002) lower than in the NT. This is clearly demonstrated in Figure 2

, where paired comparison of PPAR*γ* expression between tumours and normal adjacent tissues from the same patients showed a significant decrease of PPAR*γ* expression in PTCs (0.17±0.09 *vs* 0.48±0.34; *P*=0.02).

### PPAR*γ* immunohistochemistry

Table 3

**Table 3:** PPAR*γ* immunohistochemistry in thyroid tumours

	**PPAR*γ* immunostaining**		
**Tissue**	**Negative**	**Positive**	***P*-value**
NT (*n*=47)	11 (23%)	36 (77%)	
NH (*n*=28)	8 (29%)	20 (71%)	NS
FTA (*n*=86)	33 (38%)	53 (62%)	NS
FTC (*n*=34)	16 (47%)	18 (53%)	0.03
PTC (*n*=39)	20 (51%)	19 (49%)	0.01
PDTC (*n*=13)	13 (100%)	0	<0.0001

**Figure 3:**
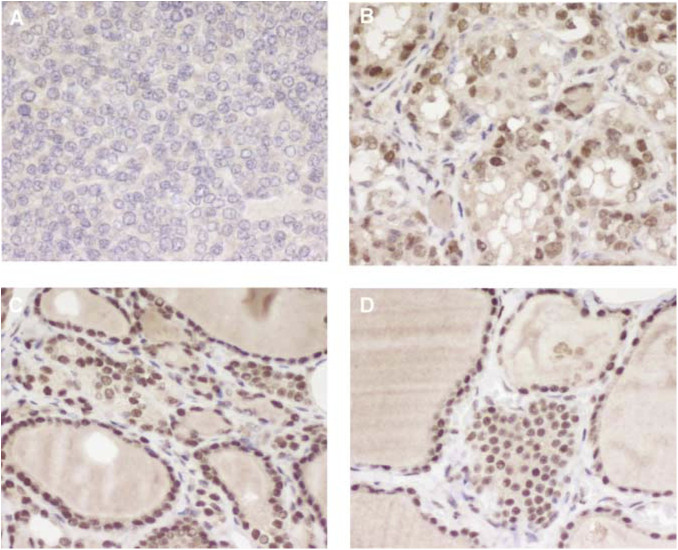
PPAR*γ* protein expression in thyroid tissues assessed by immunohistochemistry. Positive cases presented diffuse nuclear immunostaining. (**A**) Negative poorly differentiated carcinoma; (**B**) papillary carcinoma of classic variant with faint immunostaining and corresponding peritumoral (**C**) and contralateral (**D**) normal tissue, both with moderate immunoreativity. (**A–D**) original magnification × 400.

NS=not significant; NT=normal thyroid; NH=nodular hyperplasia; FTA=follicular thyroid adenoma; FTC=follicular thyroid carcinoma; PTC=papillary thyroid carcinoma; PDTC=poorly differentiated thyroid carcinoma; PPAR=peroxisome proliferator-activated receptor.

presents the intensities of PPAR*γ* nuclear immunostaining for each tumour histotype. Representative results are shown in Figure 3

. Diffuse nuclear immunoreactivity, usually faint or moderate, was detected in 77% (36 of 47) of NT tissues, 53% (18 of 34) of FTCs (*P*=0.03 *vs* normal tissue), 49% (19 of 39) of PTCs (*P*=0.01 *vs* normal tissue), 62% (53 of 86) of FTAs (*P*=0.09 *vs* normal tissue) and in 71% (20 of 28) of NHs (*P*=0.78 *vs* normal tissue). All PDTCs (*n*=13) were negative (*P*<0.0001 *vs* normal tissue). All translocation-positive FTAs (*n*=6) and five of six FTCs showed PPAR*γ* staining usually moderate or strong in intensity. Tumour staining was similar to the intensity in the normal adjacent tissue in 62% (29 of 47) of cases, was lower in 28% (13 of 47) of cases and stronger in 10% (five of 47) of cases. Frozen tissue was available in two out of the five cases staining stronger than the normal tissue; PAX8-PPAR*γ* fusion gene was present in these two cases.

### Correlation between PPAR*γ* immunohistochemistry and clinical/pathological data

Clinical and pathological features of malignant tumours are presented in Tables 4

**Table 4:** Clinical and pathological features and PPAR*γ* expression in follicular carcinomas

	**PPAR*γ* immunohistochemistry**			
**FTC**	**All (*n*=34)**	**Negative (*n*=16)**	**Positive (*n*=18)**	***P*-value**
Age at diagnosis (years±s.d.)	55.6±16.8	59.4±15.7	52.2±17.5	NS
F/M ratio	2.4/1	4.3/1	1.6/1	NS
Tumour size (cm±s.d.)	4.1±1.9	3.8±1.6	4.4±2.2	NS
*Invasiveness*				NS
Minimally invasive	26	10 (38%)	16 (62%)	
Widely invasive	8	6 (75%)	2 (25%)	
Poorly differentiated areas	7	5 (71%)	2 (29%)	NS
Local invasion	4	3 (75%)	1 (25%)	NS
Vascular invasion	24	13 (54%)	11 (46%)	NS
Distant metastasis	7	6 (86%)	1 (14%)	0.03
*Clinical status*^a^				NS
Persistent	5	4 (80%)	1 (20%)	
Remission	28	12 (43%)	16 (57%)	
Nonthyroid neoplasias	3	1 (33%)	2 (67%)	NS

PPAR=peroxisome proliferator-activated receptor; s.d.=standard deviation; NS=not significant.

a Loss of follow-up of one patient.

and 5

**Table 5:** Clinical and pathological features and PPAR*γ* expression in papillary carcinomas

	**PPAR*γ* immunohistochemistry**			
**PTC**	**All (*n*=39)**	**Negative (*n*=20)**	**Positive (*n*=19)**	***P*-value**
Age at diagnosis (years±s.d.)	41.0±19.9	38.7±18.7	43.5±21.3	NS
F/M ratio	2.5/1	1.9/1	3.4/1	NS
Tumour size (cm±s.d.)	3.7±2.4	3.9±2.1	3.5±2.3	NS
MACIS (mean±s.d.)	5.5±1.8	5.6±2.0	5.5±1.7	NS
*Class*^a^				NS
I	20	11 (55%)	9 (45%)	
II	6	3 (50%)	3 (50%)	
III	9	2 (22%)	7 (78%)	
IV	3	3 (100%)	0	
*Predominant pattern*				0.01
Classic	18	5 (28%)	13 (72%)	
Follicular	15	12 (80%)	3 (20%)	
Others	6	3 (50%)	3(50%)	
Poorly differentiated areas	6	4 (67%)	2 (33%)	NS
Local invasion	13	6 (46%)	7 (54%)	NS
Vascular invasion	9	4 (44%)	5 (56%)	NS
*Clinical status*^a^				NS
Persistent	9	3 (33%)	6 (67%)	
Remission	29	16 (55%)	13 (45%)	

PPAR=peroxisome proliferator-activated receptor; PTC=papillary thyroid carcinoma; s.d.=standard deviation; NS=not significant.

a Loss of follow-up of one patient.

. When statistical analysis was performed between PPAR*γ*-positive and -negative tumours, we observed that 86% (six of seven) of FTCs with distant metastasis were PPAR*γ* negative (*P*=0.03). There was also a trend for negative tumours to be more locally invasive (75%), to have poorly differentiated areas (71%) and to have persistent disease after surgery (80%). Most (75%) of the widely invasive FTCs were PPAR*γ* negative, whereas only 38% of minimally invasive tumours did not shown PPAR*γ* staining. The two widely invasive tumours positive for PPAR*γ* had two different components: a follicular area, which stained positive, and an insular area, which was negative. Positive PPAR*γ* staining was not correlated with age, gender, tumour size or vascular invasion. In PTC cases, we observed that 72% (13 of 18) of the tumours with a classic pattern were positive, whereas 80% (12 of 15) of the follicular variants were negative (*P*=0.01). Peroxisome proliferator-activated receptor*γ* staining did not correlate with any other prognostic variable but, interestingly, all class IV tumours (*n*=3) were negative.

## DISCUSSION

We (Marques *et al*, 2002) and others (Kroll *et al*, 2000; Nikiforova *et al*, 2002; Aldred *et al*, 2003; Cheung *et al*, 2003) have detected cases of thyroid tumours, such as FTC, FTA or PTC, that exhibit mild or moderate diffuse PPAR*γ* nuclear staining, even though they are RT–PCR negative for the PAX8-PPAR*γ* fusion gene. The question was then whether such cases present or not overexpression of PPAR*γ*. It is important to discriminate between these two possibilities, because underlying different pathogenic mechanisms may be present. For instance, if PPAR*γ* expression is found to be upregulated in PAX8-PPAR*γ*-negative tumours, it could reflect either a breakpoint between PAX8 and PPAR*γ* in a location outside the primers used in the RT–PCR reaction, or a rearrangement between PPAR*γ* and a non-PAX8 partner, or overexpression of wild-type PPAR*γ* or point mutations in the PPAR*γ* gene. The objectives of the present work were two-fold: 1 – to study, and compare, the relative expression of PPAR*γ* in the normal gland and in benign and malignant diseases of the thyroid; and 2 – to correlate PPAR*γ* immunostaining with clinical and pathological characteristics of patients with thyroid carcinomas of follicular origin. We chose to examine PPAR*γ* expression in thyroid tissues by RT–PCR, interphase FISH, real-time RT–PCR and immunohistochemistry. We first demonstrated that NT tissues express PPAR*γ* both at mRNA and at the protein level. This is in contrast with the findings by Ohta *et al* (2001), who detected PPAR*γ* mRNA in four of six PTC cell lines and in three of six PTCs, but not in NT tissues or in FTAs. However, our results are in concordance with the recent data of Aldred *et al* (2003), who have also demonstrated PPAR*γ* expression in seven of seven NT specimens. Interestingly, the mean ratio of PPAR*γ*/GAPDH mRNA obtained by the semiquantitative method of Aldred *et al* (2003) of 0.79±0.30 is not far from the ratio of 0.68±0.40 obtained by our quantitative method. As the human PPAR*γ* gene gives rise to four mRNAs, PPAR*γ*_1–4_, that differ at their 5′-end as a consequence of alternate promoter usage and splicing, and these mRNAs code two protein isoforms, PPAR*γ*_1_ and PPAR*γ*_2_, which may exert distinct biological effects, we investigated the expression of the different PPAR*γ* transcripts in the thyroid tissues. We were able to show that thyroid cells express all mRNA isoforms, but the proportion of specific variants was similar in normal tissues and in the various types of thyroid tumours studied. However, because we did not perform quantitative RT–PCR, it is possible that some tumour types predominantly express one of the isoforms. To compare PPAR*γ* expression between tumours and NT tissues, we performed quantitative analysis by real-time RT–PCR. Our assay did not distinguish wild-type transcripts from PAX8-PPAR*γ* fusion mRNAs. We observed that tumours negative for the rearrangement expressed lower levels of PPAR*γ* mRNA than NT. This was particularly evident in the PTC cases (*n*=7) in which the normal adjacent tissue of the same patient was also available for analysis (Figure 2). Upregulation of PPAR*γ* mRNA levels was found in four of the five (80%) translocation-positive tumours (three FTC and two FTA) analysed. However, we detected one PAX8-PPAR*γ*-positive case (FTC) with a PPAR*γ*/GAPDH ratio within the mean of the normal group. Interphase FISH analysis revealed that only a small subset of cells in this case harboured the translocation, which is consistent with the normal expression level of PPAR*γ* as assessed by real-time RT–PCR. Overall, there was a direct correlation between our real-time analysis of PPAR*γ* expression and the immunoreactive protein: strong immunostaining was present only in tumours with upregulated PPAR*γ* mRNA levels and mild or moderate staining was revealed in the remaining tumours, as well as in normal tissues. Notably, the translocation-positive FTC with normal PPAR*γ*/GAPDH ratio showed diffuse and faint nuclear staining. Aldred *et al* (2003) performed semiquantitative RT–PCR analysis of PPAR*γ* expression in 14 NT tissues and in 19 FTCs and also showed that nontranslocation tumours had underexpression of PPAR*γ*.

A larger number of tissues were examined by immunohistochemistry in order to determine, and compare, the prevalence of PPAR*γ* staining between normal, hyperplastic and neoplastic tissues, and to correlate staining with known prognostic variables of thyroid carcinomas. Compared to NT tissues, staining was significantly (*P*<0.05) less prevalent in FTCs, PTCs and PDTCs. This trend was also present in FTAs, although not statistically significant (*P*=0.09). Previous studies have shown that FTCs harbouring the fusion gene, hence strongly reactive with a PPAR*γ* antibody, are somewhat smaller in size (Cheung *et al*, 2003; Nikiforova *et al*, 2003), more overtly invasive, and occur at a younger age than tumours without the rearrangement (Nikiforova *et al*, 2003). In the study of French *et al* (2003), FTCs with PPAR*γ* rearrangement had vascular invasion and a solid/nested histology more frequently than translocation-negative tumours. We observed that PPAR*γ*-negative FTCs were more likely to be locally invasive, to persist after surgery, to metastasise and to have poorly differentiated areas.

We could not correlate PPAR*γ* staining with any of the prognostic variables analysed in the group of PTCs, except for tumours presenting a predominantly follicular pattern that were more often negative (80%) than classic PTCs (28%; *P*=0.01).

In summary, we have demonstrated underexpression of PPAR*γ* in PAX8/PPAR*γ*-negative thyroid tumours of follicular origin, and that a further reduction of PPAR*γ* expression is associated with dedifferentiation at later stages of tumour development.
